# Quantification of regurgitation in mitral valve prolapse with automated real time echocardiographic 3D proximal isovelocity surface area: multimodality consistency and role of eccentricity index

**DOI:** 10.1007/s10554-021-02179-2

**Published:** 2021-02-22

**Authors:** Ricardo A. Spampinato, Frank Lindemann, Cosima Jahnke, Ingo Paetsch, Florian Fahr, Franz Sieg, Maximilian von Roeder, Thilo Noack, Sebastian Hilbert, Susanne Löbe, Elfriede Strotdrees, Gerhard Hindricks, Michael A. Borger

**Affiliations:** 1 University Department for Cardiac Surgery, HELIOS Heart Center Leipzig, Strümpellstraße 39, 04289 Leipzig, Germany; 2 Department of Electrophysiology, HELIOS Leipzig Heart Center, Leipzig, Germany; 3 Department of Cardiology, HELIOS Leipzig Heart Center, Leipzig, Germany

**Keywords:** Mitral valve prolapse, Regurgitation, Echocardiography, 3D, PISA, CMR

## Abstract

Three-dimensional transthoracic echocardiography (3D-TTE) provides a semi-automated proximal isovelocity surface area method (3D-PISA) to obtain quantitative parameters. Data assessing regurgitation severity in mitral valve prolapse (MVP) are scarce, so we assessed the 3D-PISA method compared with 2D-PISA and cardiovascular magnetic resonance (CMR) and the role of an eccentricity index. We evaluated the 3D-PISA method for assessing MR in 54 patients with MVP (57 ± 14 years; 42 men; 12 mild/mild-moderate; 12 moderate-severe; and 30 severe MR). Role of an asymmetric (i.e. eccentricity index ≥ 1.25) flow convergence region (FCR) and inter-modality consistency were then assessed. 3D-PISA derived regurgitant volume (RVol) showed a good correlation with 2D-PISA and CMR derived parameters (r = 0.86 and r = 0.81, respectively). The small mean differences with 2D-PISA derived RVol did not reach statistical significance in overall population (5.7 ± 23 ml, 95% CI − 0.6 to 12; p = 0.08) but differed in those with asymmetric 3D-FCR (n = 21; 2D-PISA: 72 ± 36 ml vs. 3D-PISA: 93 ± 47 ml; p = 0.001). RVol mean values were higher using PISA methods (CMR 57 ± 33 ml; 2D-PISA 73 ± 39 ml; and 3D-PISA 79 ± 45 ml) and an overestimation was observed when CMR was used as reference (2D-PISA vs. CMR: mean difference: 15.8 ml [95% CI 10–22, p < 0.001]; and 3D-PISA vs. CMR: 21.5 ml [95% CI 14–29, p < 0.001]). Intra- and inter-observer reliability was excellent (ICC 0.91–0.99), but with numerically lower coefficient of variation for 3D-PISA (8%–10% vs. 2D-PISA: 12%–16%). 3D-PISA method for assessing regurgitation in MVP may enable analogous evaluation compared to standard 2D-PISA, but with overestimation in case of asymmetric FCR or when CMR is used as reference method.

**Electronic supplementary material:**

The online version of this article (10.1007/s10554-021-02179-2) contains supplementary material, which is available to authorized users.

## Introduction

Transthoracic echocardiography (TTE) is widely recognized as a non-invasive reference standard for quantification of organic mitral regurgitation (MR). Accurate assessment of the severity of regurgitation is of significant importance for appropriate patient management and clinical decision-making. The echocardiographic assessment of MR remains challenging. Hence, current guidelines strongly recommend an *integrative* approach using multiple qualitative, semi-quantitative, and quantitative measurements, proceeding when necessary toward quantification of effective regurgitant orifice area (EROA) and regurgitant volume (RVol) using the proximal isovelocity surface area (PISA) method [1, 2]. Notwithstanding, PISA technique has several limitations when performed by 2-dimensional (2D) echocardiography, mainly the geometric assumptions of a hemispheric flow convergence region (FCR). Recently non-gated, real-time three-dimensional (3D) color Doppler echocardiography (RT-3DE) has been introduced allowing direct automated measurement of the true PISA (3D-PISA) without geometric assumptions, showing a trend of superiority of 3D-PISA over 2D-PISA method. However, previous studies with 3D-PISA have been done in functional MR [3–5] or in mixed patient´s populations [6–8]. Data on the accuracy and utility of RT-3DE derived true PISA in the field of mitral valve prolapse (MVP) is scarce. Consequently, we sought to investigate its diagnostic usefulness for evaluation of regurgitation in MVP with direct comparison to standard 2D-echocardiography, and cardiovascular magnetic resonance (CMR), using a multi-parametric TTE approach as an independent reference method for MR severity grading.

## Material and methods

### Study population

Patients in sinus rhythm with Carpentier type II degenerative mitral regurgitation (fibroelastic deficiency, prolapse, flail leaflet, and or Barlow’s disease) were prospectively recruited to participate in an institutional review board–approved study. Between June 2018 and December 2019 a total of 58 patients with mitral valve prolapse were identified. One patient could not tolerate the supine position during CMR study and in one patient a reliable CMR study could not be obtained due to arrhythmia (multiple premature ventricular contractions). In two patients with mild MR no FCR could be identified in RT-3DE study. Finally, we evaluated 54 patients aged > 18 years, (57 ± 14 years; 42 men), with a wide spectrum of organic MR severity referred to our center for evaluation of the pathology. Exclusion criteria included organic MR due to endocarditis or rheumatic disease, previous cardiac surgery on mitral valve, concomitant aortic valve disease more than mild, intracardiac shunts, other known causes of cardiomyopathy, or typical contraindications for CMR imaging.

All baseline characteristics/clinical data were recorded at the time of the TTE examination and patients underwent CMR imaging and TTE mostly within a period of 6 h (median 120 min, IQR 64–202 min; 1 patient with an interval of 5 consecutive days). Based on a recommended integrative 2D-TTE *multiparametric* approach [1, 2] the MR was quantified and divided into 3 groups: mild/mild-moderate (MR grade 1 + /2 + , n = 12), moderate-severe (MR grade 3 + , n = 12), and severe (MR grade 4 + , n = 30). The regurgitant volume and fraction (RF) were also obtained by CMR and 3D-PISA method. Quantitative parameters were then compared between all methods.

### Standard echocardiography

Echocardiograms were performed by experienced cardiologists using commercially available ultrasound machines (Acuson SC2000 Prime, Siemens Healthcare GmbH Erlangen, Germany) equipped with 4V1 2D, 2.25–4.25 MHz transducer. TTE were acquired using the standard imaging views: parasternal long and short axes and the apical 2-, 3-, and 4-chamber views. Doppler measurements were evaluated as the average of three cycles. Evaluation of MR was carried out by an experienced echocardiographer (RS, > 10 years of experience in echocardiography with ESC certification), before RT-3DE was performed and blinded to the results of CMR exams. Color Doppler interrogation of the MR jet was performed in multiple views. Vena contracta was measured in the modified parasternal long-axis view as the narrowest portion of the jet. PISA was measured in the apical views with the lower Nyquist limit set at 32 to 42 cm/s (shifting the baseline) and zoomed in on the FCR. Peak MR jet velocity and velocity time integral (VTI) were measured using continuous-wave Doppler across the MV. PISA radius was measured approximately at the time of peak regurgitant velocity. MR volume and EROA were calculated based on the PISA measurement as recommended [1]. Efforts were made to obtain a well-defined hemispheric FCR and to avoid constraint, modifying the velocity of the aliasing contour (from 32 until to 69 cm/s) or the echocardiographic view making the regurgitation jet less eccentric and the FCR smaller and less prone to constraint. If the constraint angle (180°—alpha angle) was equal or smaller than approximately 15°, the described 8.8% inter-observer variability of the α measurements in the original publication [9], or if the radius of the proximal convergence zone was similar to the distance from the regurgitant orifice to the adjacent ventricular wall, the FCR was considered unconstrained. However, if it was not possible, angle correction was advised to improve the accuracy of EROA and RVol quantification [9]. For quantitative pulse-wave Doppler (PWD) method mitral inflow velocities were determined by PWD at the level of the mitral annulus (regurgitant valve), its diameter was measured from the apical 3-chamber view, and left ventricle outflow tract (LVOT) was used as the competent valve. LV volumes and ejection fraction (EF) were determined using the modified Simpson biplane method.

MR grading was determined using a proposed integrated evaluation with combination of structural (i.e. mitral valve morphology, left ventricle and atrial size), qualitative (i.e. color flow jet width and density), semiquantitative (i.e. vena contracta width, pulmonary vein flow, and peak E-wave velocity), and quantitative parameters (2D PISA-method and quantitative PWD). The following grading scheme was used: mild (VC < 3 mm, EROA < 20 mm^2^, RVol < 30 ml, RF < 30%), mild-moderate (VC 3–6 mm, EROA 20–29 mm^2^, RVol 30-44 ml, RF 30–39%), moderate-severe (VC 3-6 mm, EROA 30–39 mm^2^, RVol 45–59 ml, RF 40–49%), and severe (VC ≥ 7 mm, EROA ≥ 40 mm^2^, RVol ≥ 60 ml, RF ≥ 50%).

As previously observed [10], despite mid-late systolic MR versus holosystolic MR caused similar color jet area and EROA, the shorter duration of mid-late systolic MR yielded lower RVol. Consequently, absolute ERO was not linked to outcome, in contrast to RVol. Accordingly, in case of mild-late systolic MR or discrepancies between parameters, quantitative methods (i.e. regurgitant volume and fraction) were conclusive [1].

### Real time echocardiographic 3D proximal isovelocity surface area

**Fig. 1:**
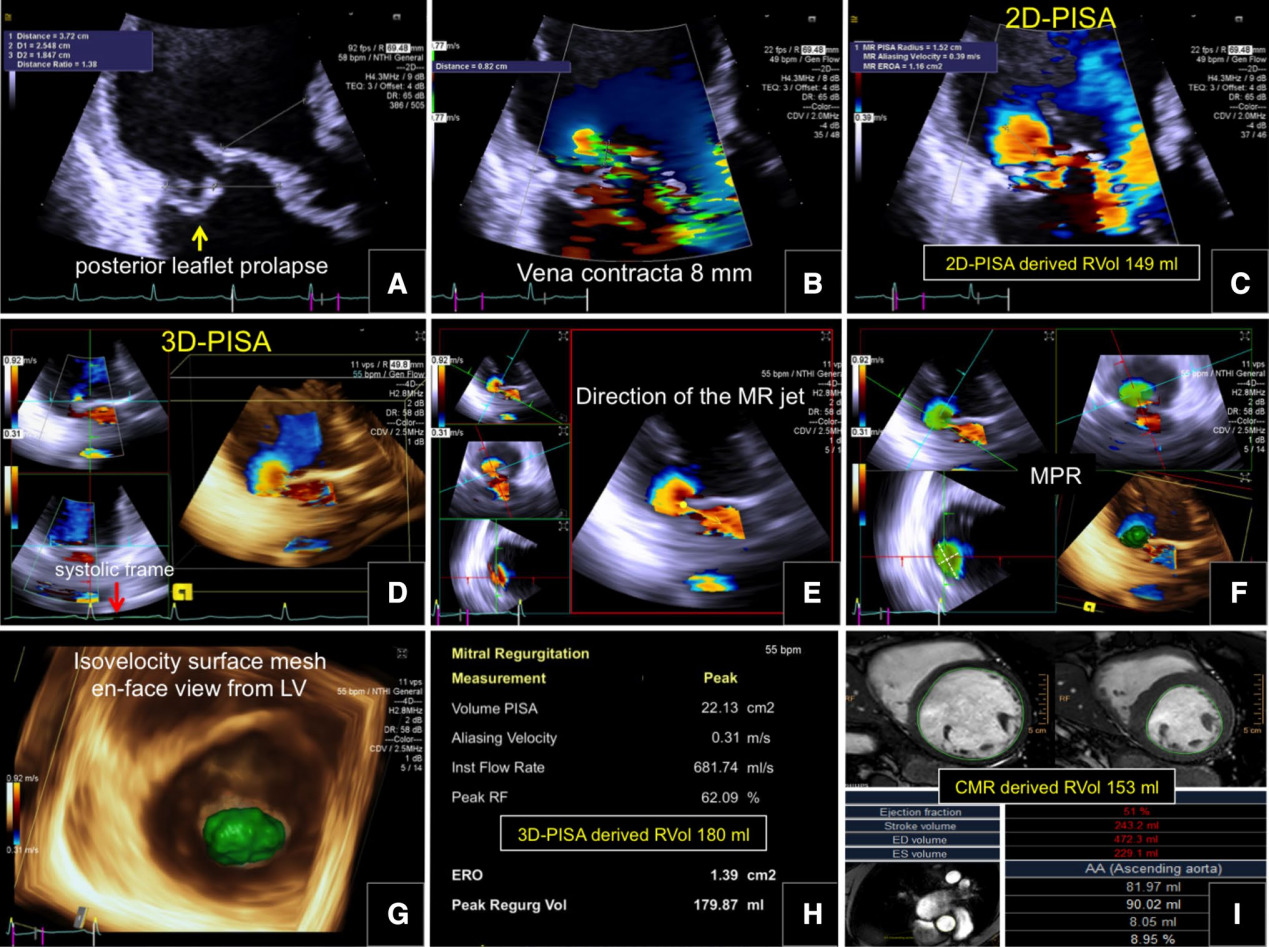
Automated algorithm to quantify the 3D-FCR derived EROA and RVol. 2D-Echocardiography depicting a posterior leaflet (P2 segment) prolapse (**a**), the vena contracta width (**b**), and 2D-PISA derived FCR (**c**). Algorithm steps: User first selects the aliasing velocity and identifies the PISA in the systolic frame with the largest systolic 3D-FCR (**d**), then the direction of the MR jet is identified (**e**) and the algorithm generates in a voxel-based 3D-space the segmentation of the true 3D-geometry of the PISA (**f**). The segmentation results are smoothened and an isovelocity surface mesh computed (**g**), note the elongated shape, and the final results reported (**h**). Cardiovascular magnetic resonance derived RVol (**i**). EROA, effective regurgitant orifice area; FCR, flow convergence region; MPR, multi-planar reconstruction; MR, mitral regurgitation; PISA, proximal isovelocity surface area; RVol, regurgitant volume; 2D-PISA, two-dimensional echocardiography derived PISA; 3D-PISA, real-time 3D echocardiography derived PISA

An ultrasound machine (Acuson SC2000 Prime, Siemens Healthcare GmbH Erlangen, Germany) equipped with a 4Z1c, 1.5–3.5 MHz transducer was used. Non-gated, real-time three-dimensional (3D) color-flow Doppler echocardiography (RT-3DE) was acquired in the apical 3-chamber view optimized for PISA with 3D B-mode and color Doppler volume sector adjusted for the mitral valve to obtain a well defined, less prone to constraint, 3D-FCR. The color Doppler box was placed to cover mitral valve. The depth and space–time settings were optimized to obtain the highest possible time resolution. Post-acquisition, offline analysis was performed at least four weeks apart from the initial evaluation, using custom software (eSie PISA Volume Analysis), which applies a previously described automated algorithm to recognize and quantify the 3D-FCR derived EROA and RVol [5, 7]. Summarizing, the user first selected the aliasing velocity and identified the PISA in the systolic frame with the largest systolic 3D-FCR. Then the direction of the MR jet was identified and the algorithm generated an isovelocity segmentation in the voxel-based 3D-space representing the true 3D-geometry (without geometric assumptions) of the PISA. The segmentation results were automatically smoothened and an isovelocity surface mesh computed (3D-FCR). Manual modifications were made to the automatically generated 3D-FCR when necessary (Fig. 1). The en-face shape of the 3D-FCR was classified, as suggested in a previous study [11], by the ratio of the two orthogonal radii (eccentricity index), as symmetrical (if the ratio was < 1.25) or asymmetrical (if the ratio was ≥ 1.25). The cutoff value of 1.25 was derived from mean value.

### Cardiovascular magnetic resonance

All CMR examinations were performed in our cardiology department on a 1.5-T MRI system (Ingenia, Philips Healthcare, Best, The Netherlands) equipped with a 28-element array coil with full in-coil signal digitalization combined with optical transmission. Image data acquisition and subsequent analysis were carried out according to current guidelines and recommendations [12]. For cine imaging, steady-state free precession (SSFP) sequences with retrospective gating were used during repetitive breath-holding. All standard cardiac geometries were acquired (multiple, gapless short-axis slices covering the entire left ventricle and 2-, 3- and 4-chamber views). Reconstructed in-plane spatial resolution was 1.3 × 1.3 mm^2^ with a slice thickness of 8.0 mm; typical temporal resolution of cine SSFP sequences was < 30 ms depending on heart rate. In addition, two-dimensional phase-contrast flow measurements were performed in the ascending aorta with the imaging plane being placed approximately 10 mm above the aortic valve and carefully positioned perpendicular to the flow direction. To avoid aliasing, velocity encoding was individually adapted, starting at 200 cm/s, and if aliasing occurred, the maximum velocity was increased by 50 cm/s steps and flow measurements were repeated accordingly. Image data acquisition was gated to the ECG signal with an in-plane spatial resolution of 1.4 × 1.4 mm^2^ and a temporal resolution of 35 phases per cardiac cycle being acquired during a 12–15 s breath-hold. Through-plane phase-contrast derived measurement were: aortic stroke volume (AoSV), aortic systolic forward flow volume (AoFF), and aortic diastolic backward flow volume (AoBF). Cine short axis images were used to measure LV end-diastolic volume (LVEDV) and LV end-systolic volume (LVESV) based on the disc summation method. LV stroke volume (LVSV) and LV ejection fraction (LVEF) were calculated accordingly. Mitral valve RVol, and RF were then calculated as follows:

$$\begin{gathered} {\text{RVol }}\left( {{\text{ml}}} \right) \, = {\text{ LVSV }} - {\text{ AoFF}} \hfill \\ {\text{RF }}\left( \% \right) \, = {\text{ RVol }} \times {1}00/{\text{LVSV}} \hfill \\ \end{gathered}$$

### Statistical analysis

Data are presented as mean (SD), median (25th to 75th percentile), or frequency (percent) as appropriate. Statistical differences were assessed using Student’s t-test for normally distributed residuals or Mann–Whitney and Wilcoxon test for non-normal variables. Fisher’s exact test was used for assessing independence/dependence in categorical variables. Multigroup comparisons of continuous variables were performed using an analysis of variance (ANOVA). Pearson correlation coefficient, Bland–Altman plots, and intraclass correlation coefficient (ICC) were used to assess correlations and agreements between methods. Rate of agreement for MR grading was evaluated by calculating a κ-statistics. In the first 25 patients, the inter- and intra-observer percentage of variation on 2D and 3D-PISA derived RVol were determined by analysis of the deviation between (re)-measurements divided by the mean of both measurements. Additionally, the mean absolute difference with the 95% confidence interval and the ICC were also determined. Two-tailed p-values < 0.05 were considered statistically significant. Analyses were performed using SPSS software (IBM-SPSS Statistics, Version 20, IBM Corp.). The study was conducted in accordance with the Declaration of Helsinki, and was approved by the local research ethics committee (270–18-ek). All patients received informed consent.

## Results

Demographic and baseline patient characteristics are presented in Table 1. CMR and Echocardiographic characteristics including quantitative parameters are shown in Tables 2 and 3. Patients with severe MR had higher TTE and CMR derived LV end-diastolic volume and stroke volume compared with MR grade 1 + /2 + and 3 + , but similar LVOT or forward stroke volume values reflecting the progressively higher regurgitant volume and fraction values through the groups. Finally, they had higher values of parameters indicating increased left atrial (LA) pressure (i.e. estimated systolic pulmonary artery pressure and LA volume index).

**Table 1:** Patient’s characteristics

Age, years	57 ± 14
Male, n (%)	42 (78)
CAD, n (%)	6 (11)
Hypertension, n (%)	38 (70)
Diabetes, n (%)	6 (11)
Dyslipidemia, n (%)	22 (41)
BSA, m2	1.9 ± 0.23
NYHA I**/**II**/**III-IV, n	21/19/14
*Mitral Valve Lesion*	
Posterior leaflet, n	37
Anterior leaflet, n	3
Bileaflet, n	11
Barlow´s disease, n	3
Flail, n	27
Multiple scallops, n	22

*CAD* coronary artery disease, *BSA* body surface area. Unless otherwise specified, values are expressed as mean ± SD

**Table 2:** Multiparametric TTE classification of MR. Echocardiographic and CMR values

		All patients (54)	MR grade 1 + /2 + (12)	MR grade 3 + (12)	MR grade 4 + (30)	
*Echocardiographic parameters*						
LVEDV, ml		175 ± 67	133 ± 44^@^	140 ± 37^@^	206 ± 69	
LVESV, ml		58 ± 29	50 ± 22	44 ± 13^@^	67 ± 33	
LV-EF, %		67 ± 7	63 ± 6	68 ± 6	68 ± 6	
LVSD, mm		36 ± 7	32 ± 7^@^	33 ± 5^@^	39 ± 6	
LAVi, ml		64 ± 26	51 ± 24^@^	52 ± 21^@^	74 ± 25	
LVOT-SV, ml		72 ± 16	76 ± 12	80 ± 13	67 ± 17	
PAPs, mmHg		42 ± 19	30 ± 12^@^	33 ± 11^@^	49 ± 21	
*CMR parameters*						
LVEDV, ml		222 ± 73	174 ± 48^@^	186 ± 45^@^	255 ± 74	
LVESV, ml		86 ± 39	70 ± 36	68 ± 25^@^	99 ± 41	
LV-EF, %		62 ± 7	61 ± 9	64 ± 7	62 ± 6	
Aorta forward flow, ml		78 ± 21	81 ± 19	80 ± 16	75 ± 23	
Aorta stroke volume, ml		74 ± 20	77 ± 20	74 ± 12	72 ± 22	

Values ​​are expressed as mean ± SD. Differences reached statistical significance with: @ group “MR grade 4 + ”

*TTE* transthoracic echocardiography, *MR* mitral regurgitation, *Grade 1* + */2* + mild/mild-moderate, *Grade 3* + moderate-severe, *Grade 4* + severe, *CMR* cardiac magnetic resonance, *LV* left ventricular, *EDV* end-diastolic volume, *ESV* end-systolic volume, *EF* ejection fraction, *LVSD* LV end-systolic diameter, *LAVi* left atrial volume index, *LVOT* left ventricle outflow tract, *PAPs* estimated systolic pulmonary artery pressure

**Table 3:** Mitral regurgitation quantitative parameters

		All patients (54)	MR grade 1 + /2 + (12)	MR grade 3 + (12)	MR grade 4 + (30)
*CMR parameters*					
LV-SV, ml		136 ± 39	103 ± 23^@^	117 ± 23^@^	156 ± 37
RVol, ml		57 ± 33	22 ± 16^@#^	37 ± 11^@^	80 ± 26
RF, %		41 ± 17	22 ± 14^@#^	33 ± 7^@^	52 ± 11
*2D-TTE parameters*					
LV-SV, ml		117 ± 44	83 ± 26^@^	96 ± 30^@^	139 ± 41
Vena contracta, mm		0.62 ± 0.21	0.30 ± 0.15^@#^	0.60 ± 0.09^@^	0.76 ± 0.09
E wave, m/s		1.36 ± 0.37	0.95 ± 0.16^@#^	1.23 ± 0.16^@^	1.58 ± 0.31
EROA, cm^2^ **		0.50 ± 0.28	0.18 ± 0.14^@#^	0.36 ± 0.09^@^	0.68 ± 0.22
RVol, ml **		73 ± 39	22 ± 16^@#^	54 ± 8^@^	101 ± 26
RF, % **		47 ± 18	21 ± 11^@#^	40 ± 4^@^	59 ± 9
*3D-PISA parameters*					
EROA, cm^2^ ***		0.54 ± 0.34	0.21 ± 0.17^@#^	0.43 ± 0.19^@^	0.72 ± 0.32
RVol, ml ***		79 ± 45	27 ± 19^@#^	65 ± 28^@^	105 ± 37
RF, % ***		48 ± 18	23 ± 13^@#^	44 ± 8^@^	60 ± 11
*PW Doppler parameters*					
MV-SV, ml		140 ± 41	100 ± 25^@#^	124 ± 16^@^	163 ± 38
RVol, ml		69 ± 42	23 ± 18^@#^	48 ± 13^@^	96 ± 36
RF, %		46 ± 18	21 ± 13^@#^	38 ± 7^@^	58 ± 11

Other abbreviations as in Table 2. Values ​​are expressed as mean ± SD. ** 2D-PISA and *** 3D-PISA derived parameters. Differences reached statistical significance with: # group “MR grade 3 + ” and @ group “MR grade 4 + ”

*SV* stroke volume, *RVol* regurgitant volume (MR), *RF* regurgitant fraction (MR), *2D* two-dimensional, *3D-PISA* real-time three-dimensional full volume color-flow Doppler derived PISA (Proximal Isovelocity Surface Area), *PW* pulse-wave, *EROA* effective regurgitant orifice area, *MV* mitral valve

Thirty-six patients underwent mitral valve repair. In whom, the initial TTE evaluation showed a 4 + MR in 28, a 3 + MR in 7, and a 2 + MR in one (this patient was in NYHA class II and showed an increase in MR severity and systolic pulmonary artery pressure > 60 mmHg on exercise echocardiography). All patients with a moderate to severe MR were symptomatic.

### Agreement between methods

**Fig. 2:**
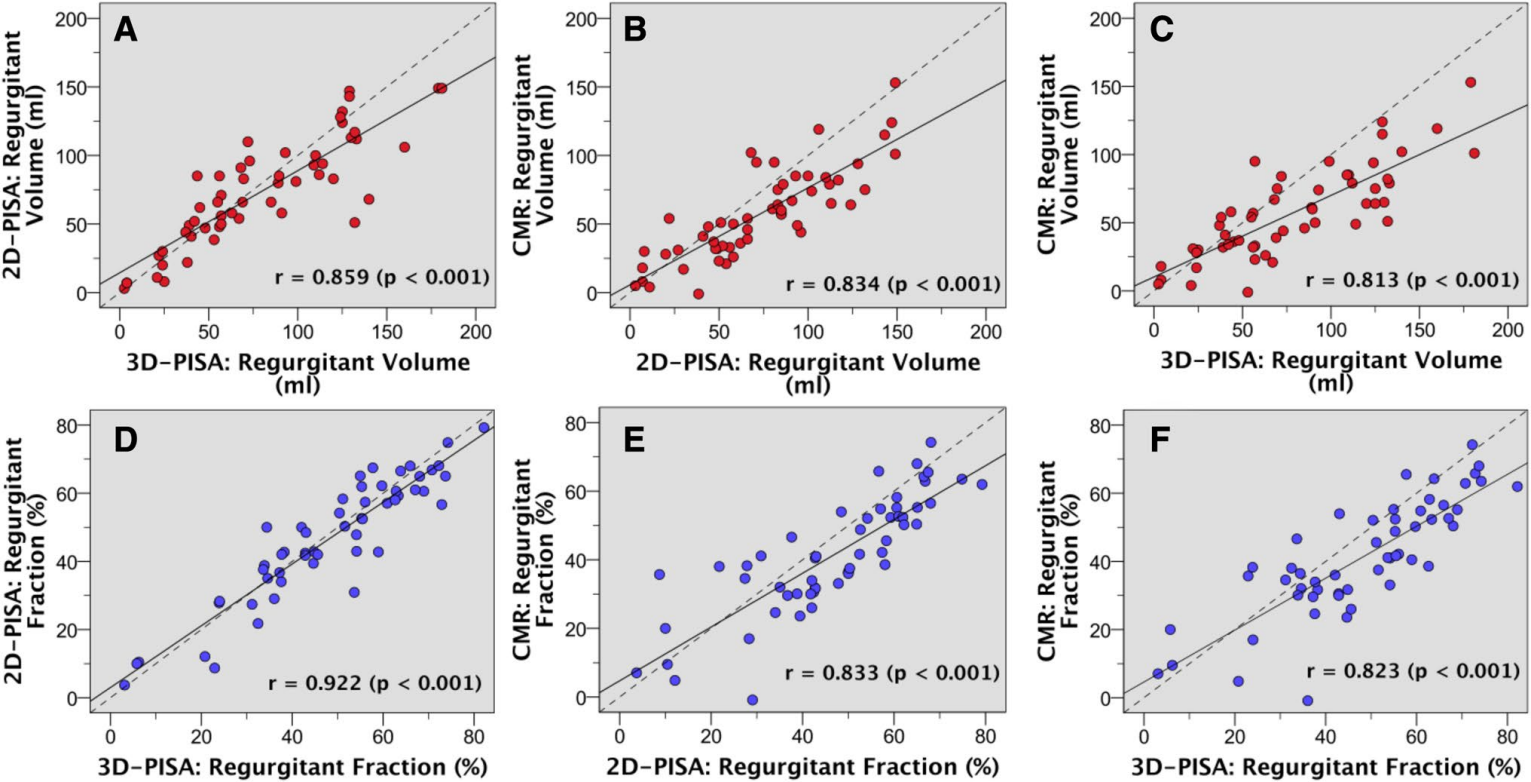
Correlation for regurgitant volume (red dots; **a**, **b**, and **c**) and fraction (blue dots; **d**, **e**, and **f**) measurements with 2D-echocardiographic flow convergence method (2D-PISA), RT-3DE derived PISA method (3D-PISA), and cardiac magnetic resonance (CMR). Dashed line indicates line of identity and solid line, linear regression. Pearson correlations (r) between methods are showed

**Fig. 3:**
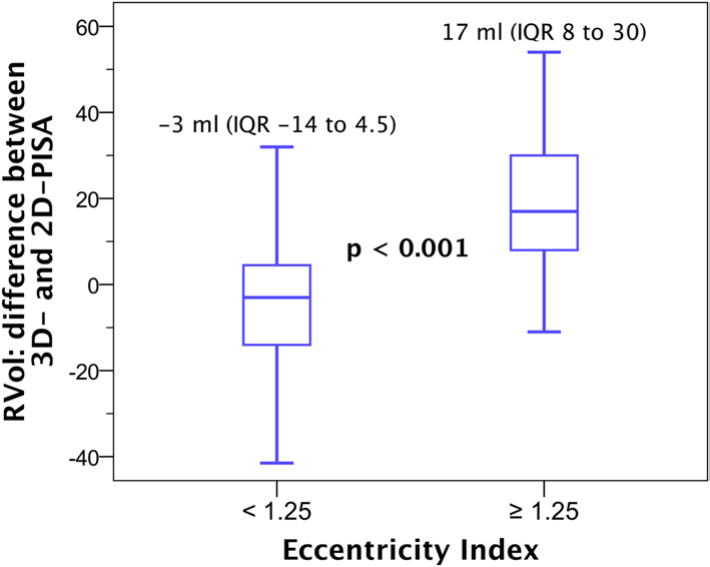
Difference in regurgitant volume (RVol) by 2D-PISA and 3D-PISA methods according to eccentricity index. The differences in RVol (RVol by 3D-PISA–RVol by 2D-PISA) were more prominent in patients with asymmetric flow convergence region (eccentricity index ≥ 1.25). Values are expressed as median (interquartile range)

**Fig. 4:**
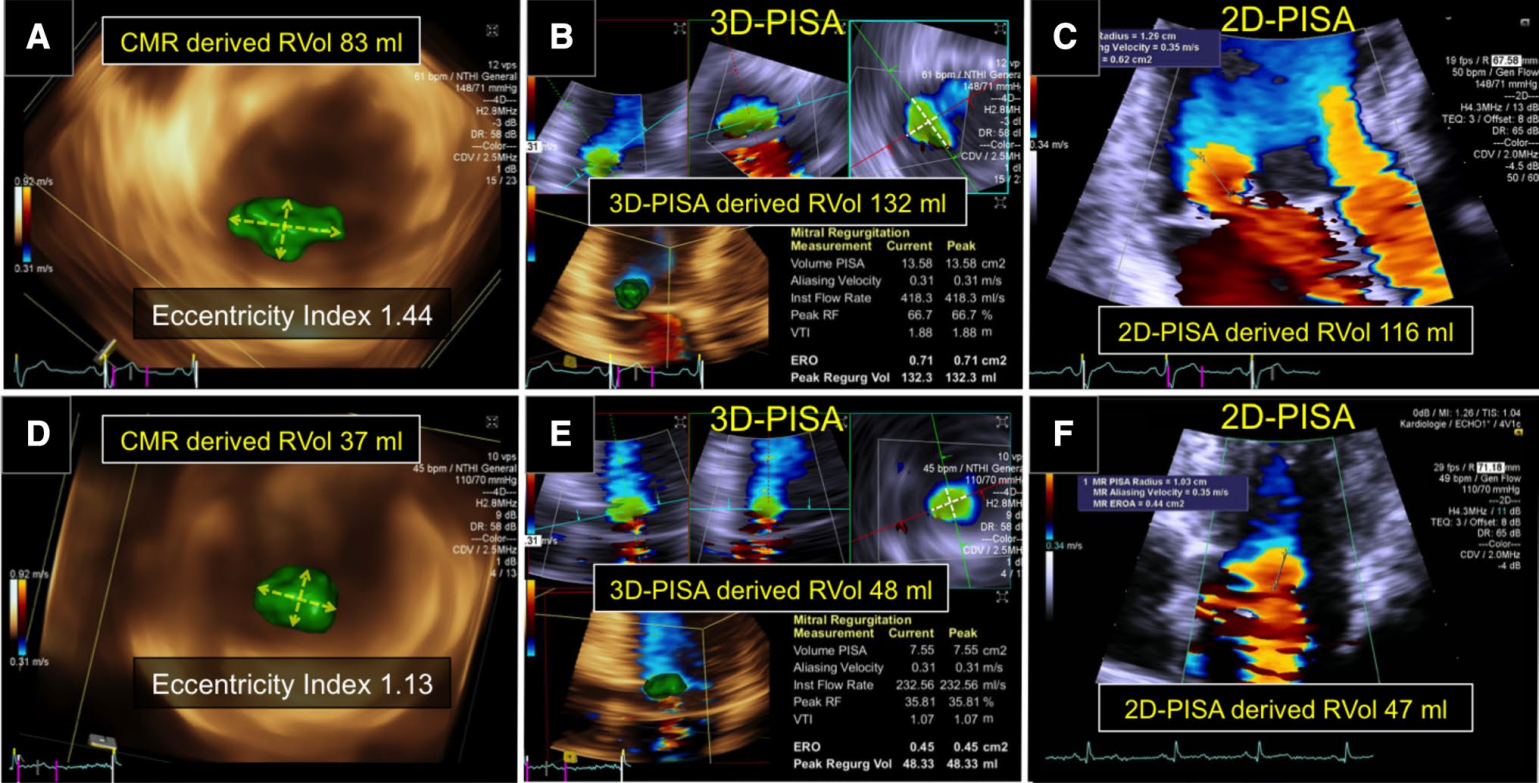
Difference in RVol by 2D-PISA and 3D-PISA methods according to eccentricity index. Clinical cases: (**a**, **b**, and **c**) example with an asymmetric 3D-FCR showing a difference in RVol between 3D-PISA and 2D-PISA of 16 ml. **d**, **e**, and **f** example of a late-systolic mitral regurgitation with a symmetric 3D-FCR showing no significant difference in RVol between methods. **a** and **d**, isovelocity surface mesh en-face from left ventricle depicting the ratio (eccentricity index) between two orthogonal radii (yellow arrow). **b** and **e**, 3D-PISA measurements from multi-planar reconstruction. **c** and **f**, 2D-PISA measurements from apical views. Abbreviations as in Fig. 1

**Fig. 5:**
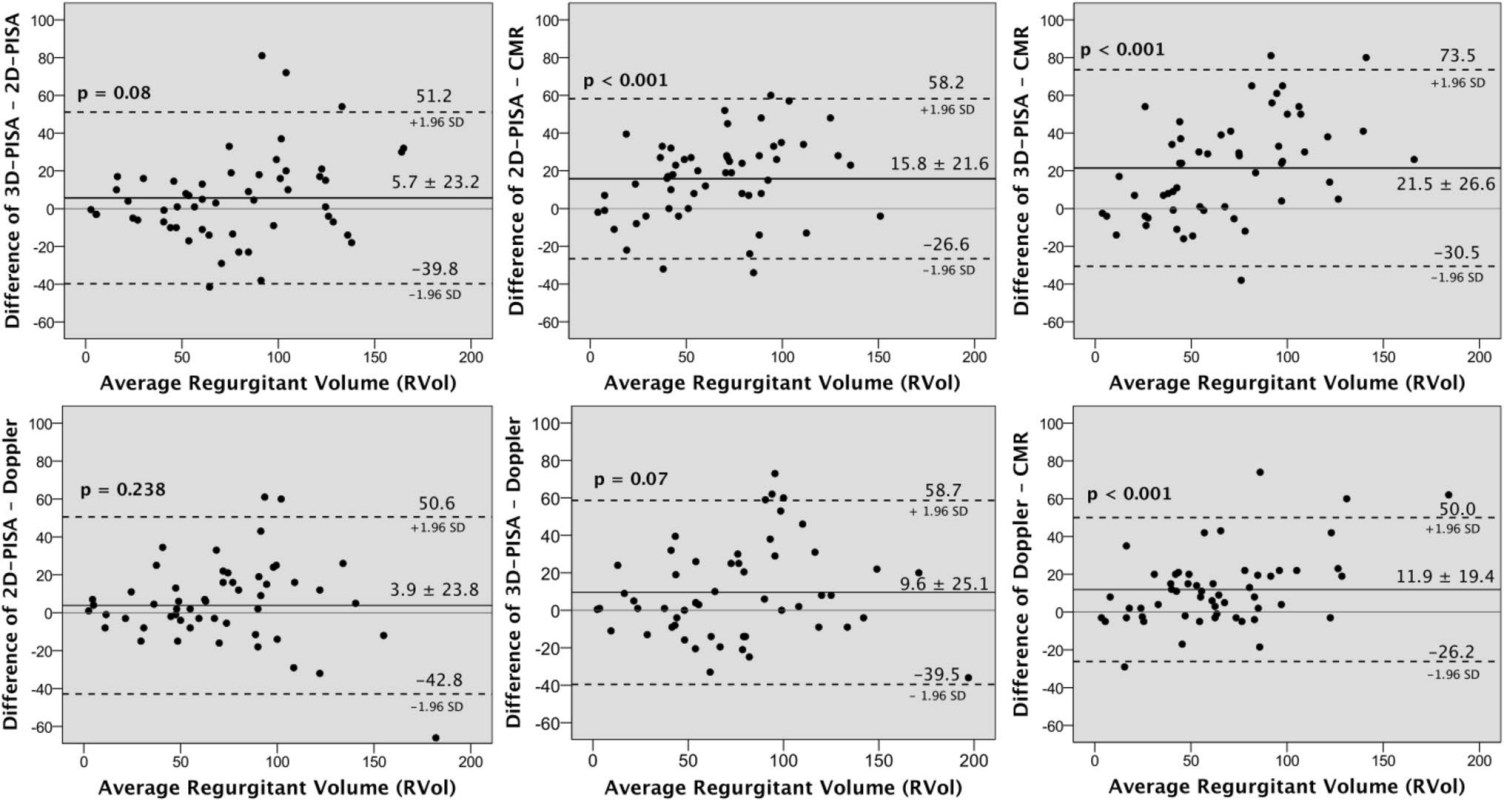
Bland–Altman plots for the agreement of measurements of regurgitant volume (RVol) by 2D-echocardiographic flow convergence method (2D-PISA), pulse-wave Doppler volumetric method (Doppler), RT-3DE derived PISA method (3D-PISA), and cardiac magnetic resonance (CMR), in patients with mitral valve prolapse

In the whole cohort the RVol and RF measurements assessed by 3D-PISA method, 2D-PISA, and CMR imaging were strongly correlated (Fig. 2). The small mean difference between 3D-PISA and 2D-PISA derived RVol (of 5.7 ml) showed statistically a tendency to higher 3D-PISA derived values (p = 0.08). Differences were more prominent in the group with asymmetric FCR (n = 21; 72 ± 36 ml by 2D-PISA and 93 ± 47 ml by 3D-PISA; p = 0.001; r = 0.87), but not significant in case of symmetric FCR (n = 33; 74 ± 41 ml by 2D-PISA and 70 ± 42 ml by 3D-PISA; p = 0.126; r = 0.93). These data are depicted in Fig. 3 and representative cases are demonstrated in Fig. 4. Notwithstanding, overall Bland–Altman plots showed good limits of agreement between 2D-PISA and 3D-PISA methods (Fig. 5), and using a recommended integrative TTE *multiparametric* approach the level of agreement for grading MR severity was substantial (k = 0.609, p < 0.001); 42 of 54 patients (78%) had the same MR grade. Nine patients were upgraded and three were downgraded only one grade scale (online resource 1).

Nonetheless, the mean RVol values assessed by 2D-PISA and 3D-PISA were significantly higher compared with CMR (73 ± 39 ml, 79 ± 45 ml, and 57 ± 33 ml, respectively; p < 0.001). Indeed, an overestimation with TTE methods was observed when CMR was used as the reference for RVol values (2D-TTE vs. CMR: mean difference 15.8 ml [95% CI 9.9 to 21.7, p < 0.001]; and 3D-PISA vs. CMR: mean difference 21.5 ml [95% CI 14.2 to 28.7, p < 0.001]; Fig. 5). Overestimation with both TTE methods was more pronounced in the groups with MR grade 3 + and 4 + and, in case of asymmetric FCR, only when 3D-PISA was compared (asymmetric FCR, n = 21: 2D-TTE vs. CMR: mean difference 9.8 ml [95% CI − 0.7 to 20.3, p = 0.064]; and 3D-PISA vs. CMR: mean difference 31.4 ml [95% CI 19.5 to 43.2, p < 0.001], Table 4 and online resource 2).

**Table 4:** Mitral regurgitant volume (RVol) and fraction (RF) measurements by CMR, 2D-, and 3D-echocardiography according eccentricity index

Eccentricity index		< 1.25 (n = 33)			> 1.25 (n = 21)		
		Mean value			Mean value		
CMR	RVol RF	55 ml 40%			62 ml 43%		
2D-PISA	RVol RF	74 ml 47%			72 ml 46%		
3D-PISA	RVol RF	70 ml 46%			93 ml 51%		

		r value	Mean difference	p value	r value	Mean difference	p value
2D-PISA vs. CMR	RVol RF	0.877	19.6 ml 7%	< 0.001 < 0.001	0.790	10 ml 2.6%	0.064 0.297
3D-PISA vs. CMR	RVol RF	0.801	15 ml 6%	0.002 0.005	0.843	31 ml 8.5%	< 0.001 < 0.001
2D-PISA vs. 3D-PISA	RVol RF	0.926	4.4 ml 1%	0.126 0.238	0.867	− 21.6 ml − 6%	0.001 0.001

*CMR* cardiac magnetic resonance, *2D-PISA* 2-dimensional proximal isovelocity surface area, *3D-PISA* 3-dimensional real-time full-volume Doppler echocardiography derived PISA, *RVol* regurgitant volume, *RF* regurgitant fraction

Discrepancies between methods were not associated with the complexity of the mitral valve lesion, single segment lesion vs. complex lesion (i.e. two or more scallops, bileaflet, or Barlow´s disease, online resource 3). Doppler volumetric method performed comparably to standard 2D echocardiography, but with numerically lower overestimation when comparing to CMR volumetric method (Fig. 5).

### Reproducibility of RT-3DE method

The intra-observer coefficients of variation for analysis of RVol by 3D-PISA method was 8 ± 7%, with a mean difference of 0.9 ml (95% confidence interval − 1.4 to 3.2; p = 0.441), an absolute mean difference of 4.5 ml (95% CI 3.2 to 5.9), and a good intraclass correlation coefficient (ICC: 0.995, 95% CI 0.989–0.998; p < 0.001; Table 5 and online resource 4). The inter-observer coefficients of variation for analysis of RVol by 3D-PISA method was 10 ± 8%, with a mean difference of 2.1 ml (95% CI − 1.1 to 5.3; p = 0.187), an absolute mean difference of 6.4 ml (95% CI 4.4 to 8.3), and a good ICC (0.991, 95% CI 0.980–0.996; p < 0.001). The inter- and intra-observer variability of RVol values assessed by 2D-PISA method are also summarized in Table 5 and depicted graphically in online resource 4.

**Table 5:** Regurgitant Volume Measurement Variability

	Bland–Altman*	ICC^†^	CV, %
*RT-3DE derived PISA method*			
Intra-observer	0.9 (12 to − 10)	0.995	8
Inter-observer	2.1 (17 to − 13)	0.991	10
*2D-PISA method*			
Intra-observer	0.8 (21 to − 19)	0.975	12
Inter-observer	3.8 (44 to − 36)	0.912	16

*RT-3DE* real-time three-dimensional full volume color-flow Doppler echocardiography, *PISA* proximal isovelocity surface area, *ICC* intraclass correlation coefficient, *CV* coefficient of variation

^*^Mean difference (2-sided 95% confidence limits of agreement). † All P < 0.001

## Discussion

The present study demonstrated that a semi-automated RT-3DE derived PISA method for assessing regurgitation in MVP may enable analogous evaluation compared to standard 2D-TTE, but with overestimation in case of asymmetric FCR or when CMR was used as an independent reference method.

Although, previous studies have already shown similar results when comparing CMR and echocardiography techniques in patients with degenerative MR [13, 14], only standard 2D echocardiographic methods has been compared.

Initial validation animal studies and first clinical experiences [6, 15] using RT-3DE evaluation of the flow convergence zone for quantification of mitral regurgitation demonstrated that 3D-PISA method is feasible and accurate. Nevertheless, subsequent studies have shown that 2D-PISA underestimates RVol when compared with 3D-PISA [7] . This could be explained due to the inherent geometric assumption of a hemispheric FCR of the 2D-method, particularly discrepant in patients with an elongated, non-circular vena contracta area (VCA). Choi and colleagues found in 211 patients with MR of mixed etiology (47% functional MR) that MR severity, asymmetrical regurgitant orifice, and eccentric jet were predictors of significant discrepancy between 3D-PISA and 2D-PISA derived RVol using CMR as reference, which was particularly the case of functional MR (FMR). Matsumura et al. evaluated 27 patients with FMR and 27 patients with organic MR and found that en-face 3D color Doppler images showed an elongated and curved PISA geometry along the leaflet coaptation in FMR, whereas the geometry was rounder in organic MR. Compared with 3D-PISA, the 2D-PISA method with the maximum radius underestimated the EROA (by 24%) in FMR, but not in organic MR. In accordance with this, we observed small not significant differences between 2D-PISA and 3D-PISA derived RVol in the overall population, but statistically significant in those with asymmetric FCR. Thus, our data elucidated an important statement in the assessment of regurgitation in MVP. Those cases with an asymmetric FCR (up to 39% of our cohort), exhibited discordance between 2D- and 3D-PISA methods as described for FMR. Nevertheless, in these patients with MVP and asymmetric FCR, when CMR was used as an independent reference method, a tendency to overestimation of the RVol with 2D-PISA was observed, rather than underestimation like in FMR. There is some data suggesting that PISA method might overestimate the regurgitant volume and fraction in “organic” MR when compared with CMR [13, 14, 16].

Some technical issues could be associated with these discrepancies in patients with MVP. First, a geometric correction factor had demonstrated that largely eliminates overestimation caused by flow constraint with the proximal convergence method [9]. In our cohort, only four patients (all with posterior mitral leaflet prolapse and consecutive severe MR) were found to display relevant constraint and accordingly angle correction was performed. Which is a lower rate of proximal flow constraint as previously reported [9]. Nevertheless, the mean difference in RVol of 15.8 ml (95% CI 9.9 to 21.7) in our study between 2D-PISA and CMR volumetric method was similar to that showed between 2D-PISA and thermodilution method after angle correction in the original study from Pu and collages (15.5 ± 19.3 ml). Suggesting that proximal flow constraint was largely corrected. Notwithstanding, for the 3D-PISA method we did not perform any angle correction, due to lack of a validated method for angle correction in case of constraint. Nevertheless, we could speculate that correcting the angle in those four patients would not modify significantly the results of the present study. Second, and beyond angle correction, 3D-PISA can be obtained from RT-3DE datasets using FCR width, length, and radius for the calculation of the hemielliptic PISA, [17] showing a better correlation and agreement with CMR imaging compared with a hemispheric PISA formula [18]. Moreover, to overcome the limitations of a formula based analysis of the FCR, the true 3D-PISA shape manually reconstructed has been proposed. Ashikhmina et al. confirmed in patients with FMR that manual reconstruction of 3D-FCR without geometric assumptions provides significantly larger EROA not only compared to conventional hemispheric PISA, but also compared to 3D based hemielliptic PISA [3]. We used custom software for a semi-automated 3D based reconstruction of the true FCR, which could result in higher RVol values. In line with this, Thavendiranathan et al. evaluated 30 patients with FMR and found that automated true 3D-PISA overestimated the RVol compared with CMR imaging. Nevertheless, we observed agreement between 3D- and 2D-PISA methods in those cases with symmetric FCR, suggesting that discrepancies with CMR derived RVol are not associated with a geometric assumption or a true 3D reconstruction of the FCR, but with another intrinsic technical issue of the flow convergence method. Third, Thavendiranathan et al. also compared the diagnostic accuracy of RT-3DE in assessing FMR using the largest systolic 3D-PISA referred as the peak 3D-PISA derived RVol and the integrated 3D-PISA derived RVol (calculated for each systolic frame) taking into account the dynamic of the MR during systole. Interestingly, they showed that compared with CMR derived RVol (33 ± 22 ml), the integrated 3D-PISA derived RVol (34 ± 26 ml) was not significantly different; however, the peak 3D-PISA derived RVol was higher (48 ± 27 ml; p < 0.001). This could account for the overestimation observed in our study.

Interestingly, despite, 3D-PISA evaluation demonstrated higher values when compared with 2D-PISA; three patients were downgraded one grade MR scale with 3D-PISA. A plausible explanation for that is Doppler angle dependency, as demonstrated in the study from Mao et al. [19]. Basically, 3D-PISA gives an urchinoide shaped FCR due to angle dependence and underestimates compared with 2D-PISA in case of non-elongated proximal flow regions, but overestimates (or 2D-PISA underestimates) in case of elongated FCR because of the hemispheric assumption of the 2D-method. That could explain our findings in case of symmetric FCR (eccentricity index < 1.25), where we observed a tendency to underestimation with 3D-PISA (difference between 3D-PISA and 2D-PISA derived RVol: − 3 ml, IQR − 14 to 4.5 ml; Fig. 3). Indeed, all these three patients, in whom the MR severity was downgraded based on 3D-PISA values, showed a symmetric FCR with an eccentricity index < 1.25.

Our study addressed another important concern about the PISA method: the lack of reproducibility, which has limited its adoption as first line parameter for the echocardiographic grading of MR. We observed a good intraclass correlation for the analysis of intra- and inter-observer variability by 2D and 3D TTE method (Table 5), but good agreement requires not only good correlation but also small observer variability. The inter-reader coefficients of variation were numerically smaller with 3D-PISA method (10%) than with 2D-PISA (16%), comparatively better to that of 29% described in the study of Cawley and coworkers. [20] Nevertheless, the limits of agreement for analysis of RVol by 2D-PISA are still considerably broad (− 36 to 44 ml). The better inter-reader variability with 3D-PISA could be in part explained with the fact that it is a semi-automated technique. Despite better reproducibility, when comparing to CMR, the level of agreement for grading MR severity using an integrative 2D TTE approach was slightly better (k = 0.571 and 74% had the same MR grade) compared with 3D-PISA method (k = 0.461 and 69% had the same MR grade). These discrepancies are similar to that described from the group of Penicka. [13] They observed a concordant grading of MR severity with CMR and 2D-TTE techniques in 76% of the individuals. Nevertheless, CMR derived RVol showed a better performance to identify adverse outcomes. That could be in part due to the inherent lower reproducibility of the 2D echocardiographic methods.

Previous data evaluating 3D-PISA in functional MR had demonstrated potential advantage of this technique compared with 2D-PISA. In the present study, 3D-PISA method performed comparably to standard 2D-PISA when assessing patients with MVP, but with overestimation in case of asymmetric FCR and the need of additional data acquisition and post-processing time. Nonetheless, to our perspective, the present results help to understand the usefulness and limitations of different diagnostic modalities when evaluating a specific group of patients with a complex valve pathology. Moreover, 3D-PISA may exhibit some potential translational outlooks in case of MVP. It may permit, from a transthoracic study, to map out the location and en-face anatomy patterns of the proximal flow convergence region, giving a quick anatomical picture of the mitral valve prolapse complexity, potentially relevant in the preoperative planning process by mitral valve repair. In addition, despite lower level of agreement between CMR and 3D-PISA, the better inter- and intra-reader reproducibility with 3D-PISA observed in our study, may overcome in part the lower clinical predictive accuracy of 2D-TTE methods observed in previous studies [13].

Finally, development of improved automated methods, and emerging machine learning algorithms, which take into account the dynamic of the MR jet with angle correction, may improve 3D-PISA method, allowing faster post-processing analysis for crosschecking quantification in case of uncertainty. Further clinical investigation regarding the potential clinical benefit of 3D-PISA method is warranted.

### Study limitations

Our study had some limitations. Although almost all studies were performed on the same day, they were not performed simultaneously. Thus, differences in hemodynamic conditions might have resulted in different RVol values. Nevertheless, a uniform tendency of overestimation with TTE derived flow convergence methods makes this hypothesis less probable. Moreover, this reflects normal clinical practice during the evaluation of patients with MR, and may not be expected to have a major impact on the results, particularly in patients with moderate and severe MR. Second, we did not take into account the potential impact of using an integrated 3D-PISA approach instead of peak 3D-PISA, taking into account the dynamic of the MR jet. It was not the aim of the study and therefore the time resolution was not enough to allow a retrospective evaluation of this hypothesis, which should be hereafter evaluated in a prospective manner. Finally, this is a preliminary relative small study, which may have introduced some inherent selection biases. Consequently, future studies with larger number of patients will have to validate our findings, and more importantly appraise its correlation with clinical outcomes in different scenarios.

## Conclusions

The results of the present study demonstrated that 3D-PISA method for assessing regurgitation in MVP may enable analogous evaluation compared to standard 2D-TTE, but with overestimation in case of asymmetric FCR or when CMR was used as the reference method. Future studies should appraise the clinical impact of these results in different scenarios.

## Supplementary information

Below is the link to the electronic supplementary material.

Electronic supplementary material 1 (PDF 500 kb)

Electronic supplementary material 2 (PDF 250 kb)

Electronic supplementary material 3 (PDF 500 kb)

Electronic supplementary material 4 (PDF 197 kb)
