# Supplementary material for: Quantification of regurgitation in mitral valve prolapse with automated real time echocardiographic 3D proximal isovelocity surface area: multimodality consistency and role of eccentricity index
Source: Int J Cardiovasc Imaging. 2021 Feb 22;37(6):1947–59. doi: 10.1007/s10554-021-02179-2 (PMC8255267; doi:10.1007/s10554-021-02179-2)
Supplement: Supplementary file 1 — Electronic supplementary material 1 (PDF 500 kb) [file 10554_2021_2179_MOESM1_ESM.pdf]

Online Resource 1 Grading of mitral regurgitation severity: Agreement between 2D and RT-3DE derived flow convergence (PISA) methods using a multiparametric transthoracic echocardiography (TTE) approach.

	TTE			
	**Grade 1+/2+**	**Grade 3+**	**Grade 4+**	
RT-3DE #				
**Grade 1+/2+**	**9**	1		10 (18.5%)
**Grade 3+**	3	**5**	2	10 (18.5%)
**Grade 4+**		6	**28**	34 (63.0%)
	12 (22.2%)	12 (22.2%)	30 (55.6%)	**42/**54 **(78%)**

2D, two-dimensional; **RT-3DE**, three-dimensional real-time full-volume Doppler echocardiography. Upgrade (3 from mild/moderate to moderate/severe, 6 from moderate/severe to severe). Downgrade (1 from moderate/severe to mild/moderate, 2 from severe to moderate/severe). Overall agreement: # **Kappa 0.609** (p <0.001).

From: Quantification of Regurgitation in Mitral Valve Prolapse With Automated Real Time Echocardiographic 3D Proximal Isovelocity Surface Area. Multimodality Consistency and Role of Eccentricity Index. Ricardo A. Spampinato, Frank Lindemann, Cosima Jahnke, Ingo Paetsch, Florian Fahr, Franz Sieg, Maximilian von Roeder, Thilo Noack, Sebastian Hilbert, Susanne Löbe, Elfriede Strotdrees, Gerhard Hindricks, Michael A. Borger. The International Journal of Cardiovascular Imaging.
