# Supplementary material for: Quantification of regurgitation in mitral valve prolapse with automated real time echocardiographic 3D proximal isovelocity surface area: multimodality consistency and role of eccentricity index
Source: Int J Cardiovasc Imaging. 2021 Feb 22;37(6):1947–59. doi: 10.1007/s10554-021-02179-2 (PMC8255267; doi:10.1007/s10554-021-02179-2)
Supplement: Supplementary file 2 — Electronic supplementary material 2 (PDF 250 kb) [file 10554_2021_2179_MOESM2_ESM.pdf]

**Online Resource 2** Mitral regurgitant volume (RVol) measurements by CMR, 2D-, and 3D-echocardiography. Severity grading using a multiparametric transthoracic echocardiographic approach.

| | **MR grade 1+/2+** | | **MR grade 3+** | | **MR grade 4+** | |
|---|---|---|---|---|---|---|
| | **Mean difference** | **P value** | **Mean difference** | **P value** | **Mean difference** | **P Value** |
| **3D-PISA vs. 2D-PISA** | 4.4 (-0.9 to 10) | 0.097 | 11 (-5 to 27) | 0.153 | 4 (-6 to 14) | 0.411 |
| **2D-PISA vs. CMR** | 0.5 (-11 to 12) | 0.934 | 17 (9 to 25) | **0.001** | 22 (13 to 30) | **<0.001** |
| **3D-PISA vs. CMR** | 4.9 (-8 to 17) | 0.409 | 28 (12 to 44) | **0.002** | 26 (15 to 36) | **<0.001** |

**CMR**, cardiac magnetic resonance; **MR**, mitral regurgitation; **2D-PISA**, 2-dimensional proximal isovelocity surface area; **3D-PISA**, 3-dimensional real-time full-volume Doppler echocardiography derived PISA. Data are presented as mean (95% confidence interval).

From: Quantification of Regurgitation in Mitral Valve Prolapse With Automated Real Time Echocardiographic 3D Proximal Isovelocity Surface Area. Multimodality Consistency and Role of Eccentricity Index. Ricardo A. Spampinato, Frank Lindemann, Cosima Jahnke, Ingo Paetsch, Florian Fahr, Franz Sieg, Maximilian von Roeder, Thilo Noack, Sebastian Hilbert, Susanne Löbe, Elfriede Strotdrees, Gerhard Hindricks, Michael A. Borger. The International Journal of Cardiovascular Imaging.
