# Supplementary material for: Quantification of regurgitation in mitral valve prolapse with automated real time echocardiographic 3D proximal isovelocity surface area: multimodality consistency and role of eccentricity index
Source: Int J Cardiovasc Imaging. 2021 Feb 22;37(6):1947–59. doi: 10.1007/s10554-021-02179-2 (PMC8255267; doi:10.1007/s10554-021-02179-2)
Supplement: Supplementary file 3 — Electronic supplementary material 3 (PDF 500 kb) [file 10554_2021_2179_MOESM3_ESM.pdf]

Online Resource 3. Baseline characteristics and discrepancies between methods according to valve lesion complexity.

	Single scallop (32)	Complex lesion (22)
**Basic clinical characteristics**		
Age, years	60 ±11	53 ±17
Male, n (%)	26 (81)	16 (73)
NYHA I/II/III-IV, n	12/12/8	9/7/6
**Basic TTE characteristics**		
LVEDV, ml	188 ±76	157 ±47
LVESV, ml	62 ±33	52 ±20
LVOT-SV, ml	72 ±18	72 ±15
LAVi, ml	70 ±29	56 ±19[@]
PAPs, mmHg	47 ±19	35 ±17[@]
MR grade 1-2+/3+/4+, n	5/6/21	7/6/9
**Basic CMR characteristics**		
LVEDV, ml	241 ±78	193 ±55[@]
LVESV, ml	95 ±43	72 ±28[@]
Aorta forward flow, ml	80 ±21	75 ±20
**MR quantification discrepancies (RVol) ***		
2D-PISA vs. CMR, ml	16.9 (9 to 24)[#]	14.2 (4 to 24)[#]
3D-PISA vs. CMR, ml	24.7 (14 to 35)[#]	16.8 (7 to 27)[#]
3D-PISA vs. 2D-PISA, ml	7.8 (-2.2 to 18)	2.6 (-3.5 to 9)
PW Doppler vs. CMR, ml	11 (4 to 18)[#]	13 (4 to 22)[#]

Complex lesion: multiple scallops, or bileaflet, or Barlow´s disease. **TTE**, transthoracic echocardiography; **LV**, left ventricular; **EDV**, end-diastolic volume; **ESV**, end-systolic volume; **LVOT**, left ventricle outflow tract; **SV**, stroke volume; **LAVi**, left atrial volume index; **PAPs**, estimated systolic pulmonary artery pressure; **MR**, mitral regurgitation; **RVol**, regurgitant volume (MR); **2D**, two-dimensional; **3D-PISA**, real-time three-dimensional full volume color-flow Doppler derived PISA (Proximal Isovelocity Surface Area); **PW**, pulse-wave; **CMR**, cardiovascular magnetic resonance. * Values are expressed as mean difference (95% CI). Differences reached statistical significance: @ with group “single scallop” and # for differences between methods.

From: Quantification of Regurgitation in Mitral Valve Prolapse With Automated Real Time Echocardiographic 3D Proximal Isovelocity Surface Area. Multimodality Consistency and Role of Eccentricity Index. Ricardo A. Spampinato, Frank Lindemann, Cosima Jahnke, Ingo Paetsch, Florian Fahr, Franz Sieg, Maximilian von Roeder, Thilo Noack, Sebastian Hilbert, Susanne Löbe, Elfriede Strotdrees, Gerhard Hindricks, Michael A. Borger. The International Journal of Cardiovascular Imaging.
