# Supplementary material for: Quantification of regurgitation in mitral valve prolapse with automated real time echocardiographic 3D proximal isovelocity surface area: multimodality consistency and role of eccentricity index
Source: Int J Cardiovasc Imaging. 2021 Feb 22;37(6):1947–59. doi: 10.1007/s10554-021-02179-2 (PMC8255267; doi:10.1007/s10554-021-02179-2)
Supplement: Supplementary file 4 — Electronic supplementary material 4 (PDF 197 kb) [file 10554_2021_2179_MOESM4_ESM.pdf]

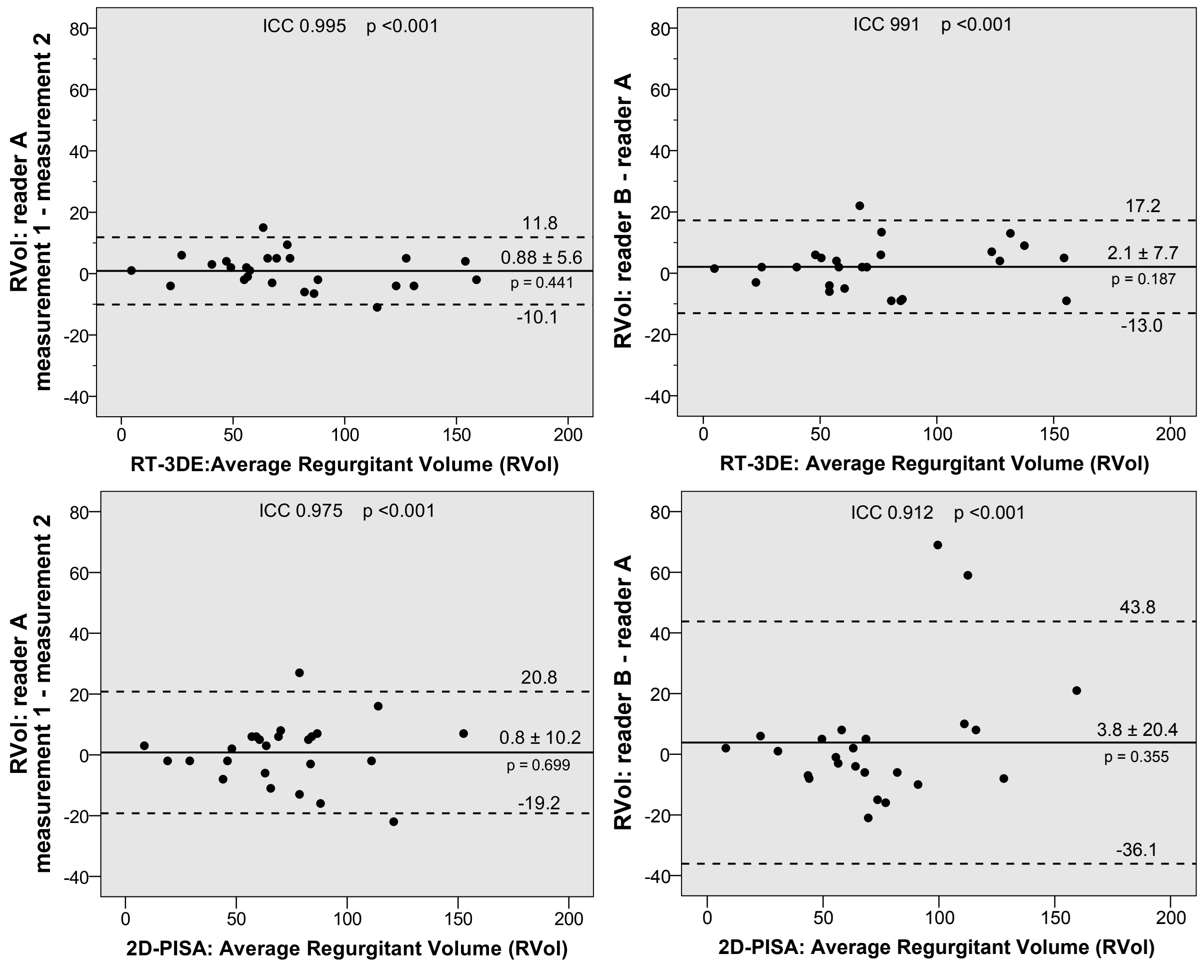

Online Resource 4- Bland-Altman plots for regurgitant volume (RVol) measurement: intra- (left panels) and inter-observer (right panels) variability with 3D-PISA (top panels) and 2D-PISA method (bottom panels). ICC = intraclass correlation coefficient.

From: Quantification of Regurgitation in Mitral Valve Prolapse With Automated Real Time Echocardiographic 3D Proximal Isovelocity Surface Area. Multimodality Consistency and Role of Eccentricity Index. Ricardo A. Spampinato, Frank Lindemann, Cosima Jahnke, Ingo Paetsch, Florian Fahr, Franz Sieg, Maximilian von Roeder, Thilo Noack, Sebastian Hilbert, Susanne Löbe, Elfriede Strotdrees, Gerhard Hindricks, Michael A. Borger. The International Journal of Cardiovascular Imaging.
